# Nitrous Oxide and Chloroform

**Published:** 1883-05

**Authors:** 


### Nitrous Oxide and Chloroform.

Nitrous oxide and ether are frequently administered in combination, or rather in succession, as a means of producing anæsthesia. The employment of a combination of nitrous oxide and chloroform has been recently advocated by M. de Saint Martin. Experiments on animals lead him to prefer the mixture of eighty-five volumes of nitrous oxide with fifteen volumes of oxygen and six or seven grammes of chloroform added to each hectolitre. This mixture causes anæsthesia very rapidly and the period of excitation is suppressed; and the chloroform, moreover, being much diluted does not irritate the air-passages. The working zone of this mixture is far greater than that of chloroform alone.

